# Different gaze strategies during eye versus hand tracking of a moving target

**DOI:** 10.1038/s41598-018-28434-6

**Published:** 2018-07-03

**Authors:** Frederic R. Danion, J. Randall Flanagan

**Affiliations:** 1 Aix Marseille University, CNRS, Institut de Neurosciences de la Timone, Marseille, France; 2 Department of Psychology and Centre for Neurosciences Studies, Queen’s University, Ontario, Canada

## Abstract

The ability to visually track, using smooth pursuit eye movements, moving objects is critical in both perceptual and action tasks. Here, by asking participants to view a moving target or track it with their hand, we tested whether different task demands give rise to different gaze strategies. We hypothesized that during hand tracking, in comparison to eye tracking, the frequency of catch-up saccades would be lower, and the smooth pursuit gain would be greater, because it limits the loss of stable retinal and extra-retinal information due to saccades. In our study participants viewed a visual target that followed a smooth but unpredictable trajectory in a horizontal plane and were instructed to either track the target with their gaze or with a cursor controlled by a manipulandum. Although the mean distance between gaze and target was comparable in both tasks, we found, consistent with our hypothesis, an increase in smooth pursuit gain and a decrease in the frequency of catch-up saccades during hand tracking. We suggest that this difference in gaze behavior arises from different tasks demands. Whereas keeping gaze close to the target is important in both tasks, obtaining stable retinal and extra-retinal information is critical for guiding hand movement.

## Introduction

Many studies emphasize an intricate relationship between eye and hand movements^1^. For instance each time we initiate a reaching movement toward a spatial location, to grasp or touch an object, this movement is typically preceded by a saccadic eye motion toward the same spatial goal^2–5^. Because pointing accuracy is altered when this saccade is prevented^5–8^, and because it is difficult to move our eyes away from the target before completion of the hand movement^9,10^, it is advocated that fixation of the target is a favored, possibly necessary requirement for accurate hand movements. The underlying assumption is that, when the eye is fixating the target, efferent and/or afferent signals related to gaze position can facilitate the transformation from a retinotopic to a body-centered frame of reference frame, thereby promoting the accuracy of hand movement^5,11–13^. Furthermore, it can be advocated that fixating the target also improves reach accuracy because it allows both the use of central vision in guiding the hand when it is close to the target, and the use of peripheral vision in providing a directional error signal^14^.

In contrast to the numerous studies that have examined the contribution of gaze when reaching to stationary targets, relatively little is known about gaze behavior and function when moving the hand towards a moving target^15^. Previous studies have characterized gaze behavior when tracking a moving target with the eyes^16–18^, or when concurrently tracking a moving target with the eyes and the hand^19–24^. In addition, previous work has examined gaze behavior when required to intercept with their hand a target moving along a predictable or unpredictable trajectory^15,25,26^. However, to our knowledge gaze behavior when continuously tracking a moving target with the hand tracking without explicit instructions about gaze (i.e., with free gaze) has not been explored.

The main objective of the current study was to investigate whether gaze behavior is driven by different strategies when the goal of the task is to track a moving target as accurately as possible with the eyes or with the hand. So far it is well established that when humans are required to track a moving target with their eyes, eye movements result from a succession of smooth pursuit episodes interrupted by catch-up saccades when position and/or velocity error increase^27,28^. As previously exposed, because fixating a static target improves reach accuracy, we reasoned that, when participants are required to track a moving target with their hand, fixating the moving target still remains advantageous. As a result we hypothesized that, even though it is crucial to monitor both eye and hand current positions for efficient hand tracking, gaze will mostly focus on the target and not the hand. Furthermore, to simultaneously limit the loss of stable retinal and extra-retinal information due to saccades^29^, while still keeping gaze close from the moving target, we hypothesized that, compared to eye tracking, hand tracking should increase smooth pursuit activity while conversely decreasing saccadic activity. Previous studies in which participants were explicitly instructed to track the target simultaneously with their eyes and their hand have shown that, in comparison to pure eye tracking, the frequency of catch-up saccades decreases and the smooth pursuit gain increases^19,23,24^. However it is not clear whether these effects are due to the demands of hand tracking per se, or arise because of the dual tracking participants are asked to perform. Furthermore we felt it was important to document natural (spontaneous) gaze behavior during hand tracking, meaning in the absence of explicit requirements for gaze^15^.

## Methods

### Participants

Eighteen self-proclaimed right-handed participants (Age: 24.2 ± 6.9 yrs., 10 female) participated in this study. None of the participants had neurological or visual disorders. They were naïve as to the experimental conditions and hypotheses, and had no previous experience of ocular motor testing. All participants gave written informed consent prior to the study. The experimental protocol was approved by the General Research Ethics Board at Queen’s University in compliance with the Canadian Tri-Council Policy on Ethical Conduct for Research Involving humans. Each experimental protocol lasted about one hour, and participants were compensated $15 for their participation.

### Apparatus

**Figure 1:**
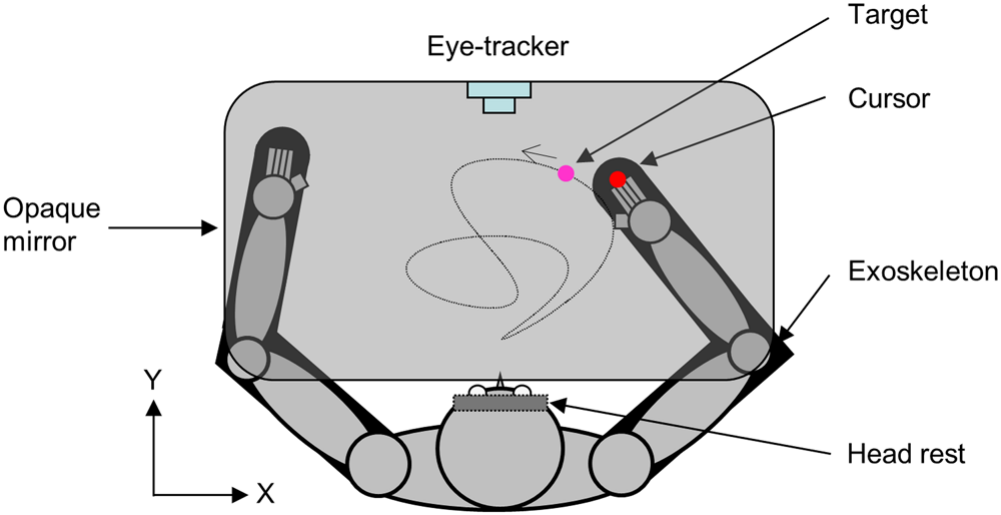
Top view of the experimental setup. Both arms of the participant were inserted into an exoskeleton. Placed above the arms, an opaque mirror blocked their view. A purple target was projected on the mirror from above and appeared at the height of the hand. The dotted line shows an example target path (not visible to the participant). During eye tracking, no cursor was displayed, both arms were immobile, and the participant had to track as accurately as possible the moving target with the eyes. During hand tracking, a red cursor representing the right index fingertip was also displayed and the participant was instructed to move his/her right arm so as to bring the cursor as close as possible to the moving target.

The experimental setup is illustrated in Fig. 1. Being comfortably seated, participants performed the tasks with of a robotic exoskeleton (KINARM, BKIN Technologies) supporting both arms in the horizontal plane while allowing flexion and extension movement at the elbow and shoulder joint^30,31^. To minimize measurement errors, participants’ head movements were restrained by a padded forehead rest so that the eyes in primary position were directed toward the center of the horizontal ‘work plane’ in which the hand moved and visual stimuli were displayed. In some trials participants controlled the position of a cursor in the work plane by moving their hand. Participants could not see their hand because the stimuli were projected from above onto an opaque mirror positioned over the arm. When the cursor was at the center of the work plane, the elbow and shoulder were comfortably positioned so that both hands lied in the participant’s mid-sagittal plane. Hand movements were recorded at a sampling rate of 1000 Hz with a resolution of 0.1 mm.

The cursor and the target were represented, respectively, as red and purple filled circles (0.6 cm in diameter). A built-in video based eye tracker (Eyelink 1000; SR Research Ltd., Canada) recorded eye movements at 500 Hz. Before the experiment, gaze position in the work plane was calibrated by having participants fixate a grid of targets. When looking at the center of the region of the work plane (and the center of target motion), a 1 cm change in gaze position corresponded to a 1.6° change in gaze angle.

### Procedure

Two experimental conditions were tested. In the first condition called EYE-TRACK participants were instructed to track as accurately as possible the target with their eyes while their hands were immobile. In the second condition called HAND-TRACK participants were instructed to track as accurately as possible the same target with a cursor representing the position of the hand in the work plane. In this condition there was no explicit requirement in terms of eye motion. During both the eye and hand tracking tasks, the motion of the target resulted from the combination of sinusoids: two along the X axis (one fundamental and a second or third harmonic) and two along the Y axis (same procedure; see Fig. 1 for axes). We used the following equation to construct target motion

1 $${x}_{t}={A}_{1x}\,cos\omega t+{A}_{2x}\,\cos ({h}_{x}\omega t-{\phi }_{x})$$

2 $${y}_{t}={A}_{1y}\,cos\omega t+{A}_{2y}\,\cos ({h}_{y}\omega t-{\phi }_{y})$$

**Figure 2:**
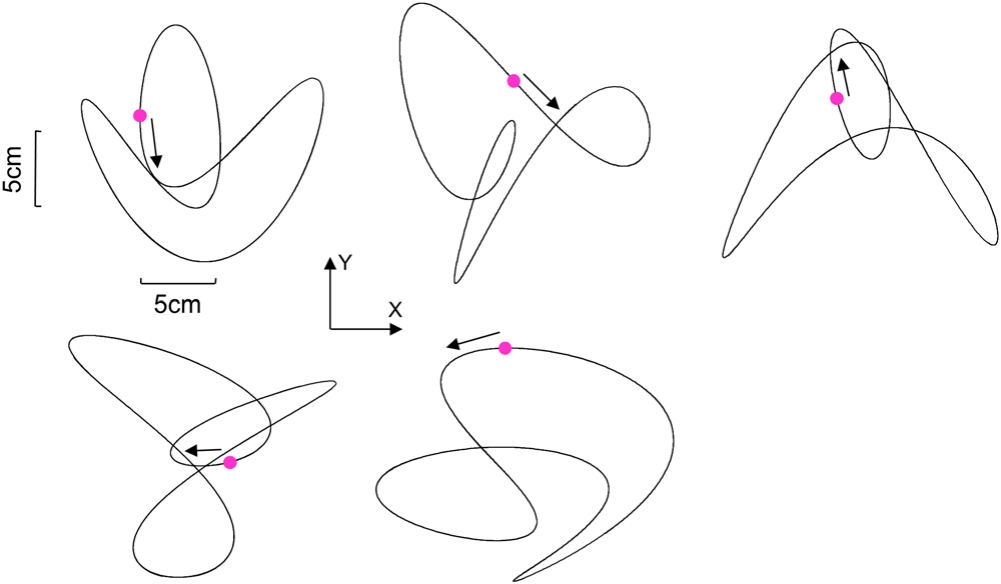
Target paths used across all the experimental conditions. The purple dot shows the initial position of the target, and the arrow shows its initial direction. The paths are shown in the horizontal work plane. All target paths had similar length (see methods for more details).

A similar technique was used elsewhere to generate pseudo-random 2D pattern while preserving smooth changes in velocity and direction^18,25^. A total of 5 different patterns were used throughout the experiment (see Fig. 2).

All trajectories had a period of 5 s (fundamental = 0.2 Hz). The parameters (gain, frequency, phase, and harmonics) used to generate all our patterns are listed in Table 1. They were selected so as to maintain the same path length over a 1 cycle (78 cm). Given that each trial was 10 s long (i.e. two cycles), the total distance covered by the target was 156 cm, resulting in a mean tangential target velocity of 15.6 cm/s.

**Table 1:** Target trajectory parameters.

Trajectory	*A1x* (cm)	*A2x* (cm)	Harmonic x	Phase x (°)	*A1y* (cm)	*A2y* (cm)	Harmonic y	Phase y (°)
1	5	5	2	45	5	5	3	−135
2	4	5	2	−60	3	5	3	−135
3	4	5.1	3	−60	4	5.2	2	−135
4	5	5	3	90	3.4	5	2	45
5	5.1	5.2	2	−90	4	5	3	22.5

Before the experimental session each participant completed a familiarization session with 5 trials in each experimental condition. Each participant then completed one block of 10 trials in each experimental condition, with half of the participants starting with the eye tracking condition first. Overall a total of 20 experimental trials were collected per participant.

### Data analysis

We first performed a sequence of analyses to separate periods of smooth pursuit, saccades and blinks from the raw eye position signals. The identification and removal of the blinks (1% of the total trial duration on average) was performed by visual inspection. Eye signals were then low-pass filtered with a 4^th^ order Butterworth filter using a cutoff frequency of 25 Hz. The resultant signals were differentiated to obtain velocity traces, and then were low-pass filtered again with a cutoff frequency of 25 Hz to remove the noise from the numerical differentiation. These eye velocity signals were differentiated to provide accelerations traces that we also low-pass filtered at 25 Hz to remove noise. The identification of the saccades was based on acceleration and deceleration peaks of the eye (>1500 cm/s²). Based on these computations, periods of pursuit and of saccades were extracted.

**Figure 3:**
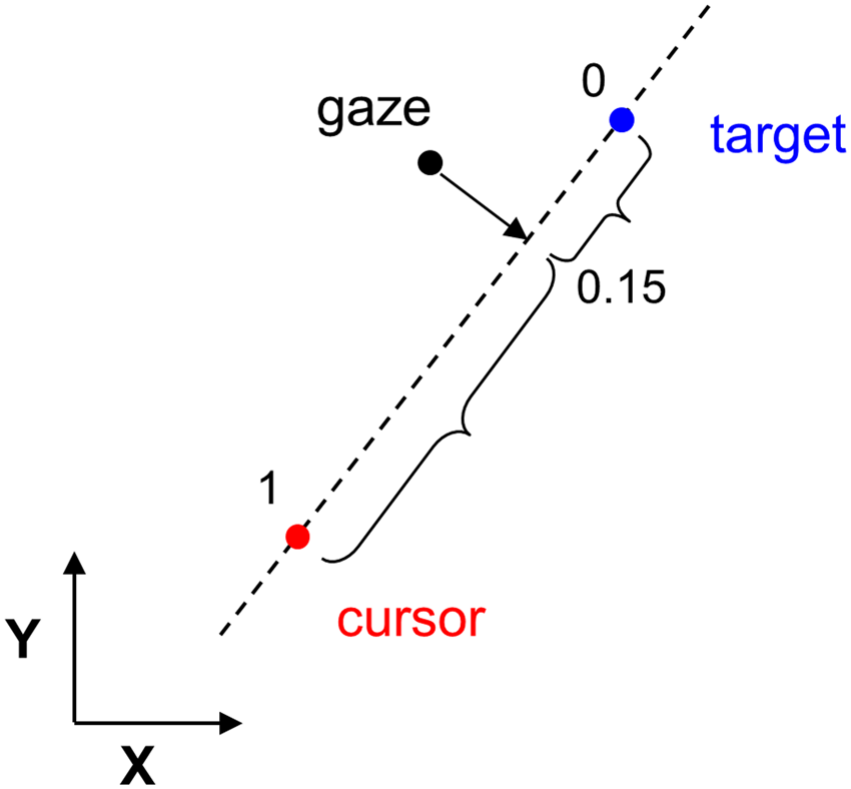
Method to estimate the relative gaze position between the cursor and target during the hand tracking task. Gaze position was projected perpendicularly onto the target-cursor axis. A normalization procedure ensured that, on this axis, target position was set at 0, and cursor position at 1.

To assess the participants’ ability to perform the tracking of target the following dependent variables were extracted from each trial. For all trials we computed the mean Euclidian distance between gaze position and target position. For each hand tracking trial, we computed the mean Euclidian distance between the cursor and target. The temporal relationship between cursor and target, and between eye and target was estimated by means of cross correlations that simultaneously took into account the vertical and horizontal axes. To simultaneously cross-correlate horizontal (x) and vertical (y) position signals between effectors, we interleaved the x and y signals, and always time shifted these interleaved signals by a multiple of two samples^32^. To assess more specifically how gaze was shared between the target and cursor during hand tracking, we developed the procedure illustrated in Fig. 3. At each point in time we projected the gaze position onto an axis connecting the target and cursor and determined the relative position along this axis, with 0 indicating that gaze projected on the target, 1 indicating gaze projected on the cursor, and a value of 0.5 indicating that gaze was equidistance between the cursor and target. For each trial, the distribution of relative gaze position was inspected.

To better characterize saccades and smooth pursuit, the following dependent variables were calculated. First we computed for each trial the mean saccade rate, that is the average number of saccades per second. Second, we computed for each saccade its amplitude, duration, and accuracy with respect to the target position (i.e., distance between gaze and target). These values were then averaged within each trial. Third, to evaluate the performance of smooth pursuit, we computed its mean tangential velocity as well as its gain (SPgain) by averaging the ratio between instantaneous gaze and target tangential velocities (only situations where target tangential velocity was greater than 10 cm/s were considered)^33^. Fourth, we calculated the mean velocity error of smooth pursuit, that is the average absolute difference between the tangential velocity of the eye and the target. Finally, to assess the relative contribution of saccades and smooth pursuit, we computed, for each trial, the total distance travelled by the eye with saccades and then expressed this as a percentage of the total distance travelled by the eye using both saccades and smooth pursuit^33,34^. For all these analyses the first second of each trial was discarded. All dependent variables were averaged across trials.

To compare gaze behavior in eye and hand tracking tasks, we used one-way ANOVA with TASK as within-subject factor (EYE-TRACK vs. HAND-TRACK) with a conventional 0.05 significance threshold.

## Results

### Typical trials

**Figure 4:**
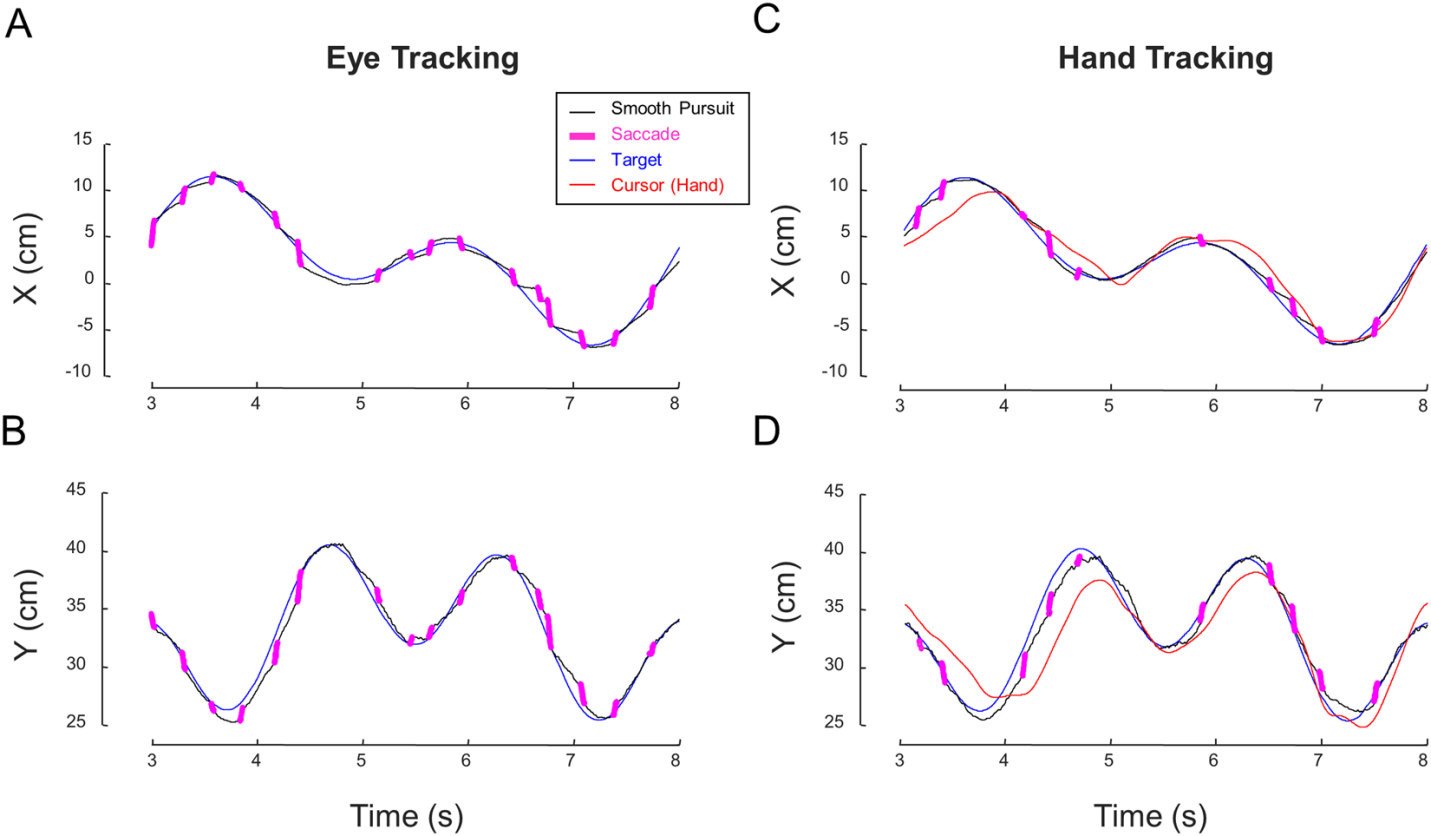
Typical trials by the same participant under each experimental condition for the same target path. A and B, Eye and target signals during the eye tracking task. The top graph presents the X component and the bottom graph the Y component. Only half of the trial (5 s) is displayed. C and D. Same as A and B for the hand tracking task. Note the smaller number of catch-up saccades under hand tracking.

Figure 4 plots representative trials, performed by the same participant and with the same target path, from the two experimental conditions. As can be seen, during both tasks gaze was always directed toward the target, as indicated by both a small delay and small distance between the eye and target positions. When tracking the target with the hand, the lag and the distance between the cursor (hand) and target were substantially larger than the lag and distance between gaze and the target, indicating that eye tracking was more accurate than hand tracking. Importantly, catch-up saccades (in magenta) occurred less frequently during hand tracking as compared to eye tracking (10 vs. 16 saccades). In the next section, we analyze in more details these observations.

### Eye vs. Hand tracking performance

Participants were more accurate in tracking the target with their eyes than with their hand. On average the mean distance between the eye and target under EYE-TRACK was 1.09 cm, whereas the mean distance between the cursor and target under HAND-TRACK was 2.47 cm, a more than two-fold difference (F(1,17) = 185.56; p < 0.001). A similar conclusion was obtained in the temporal domain. On average the eye lagged behind the target by 24 ms under EYE-TRACK, whereas the hand lagged behind the target by 108 ms under HAND-TRACK (F(1,17) = 87.96; p < 0.001). Overall we conclude that participants were more accurate in tracking the target with their eyes during the eye tracking task than with their hand during the hand tracking task.

### Overall gaze behavior

**Figure 5:**
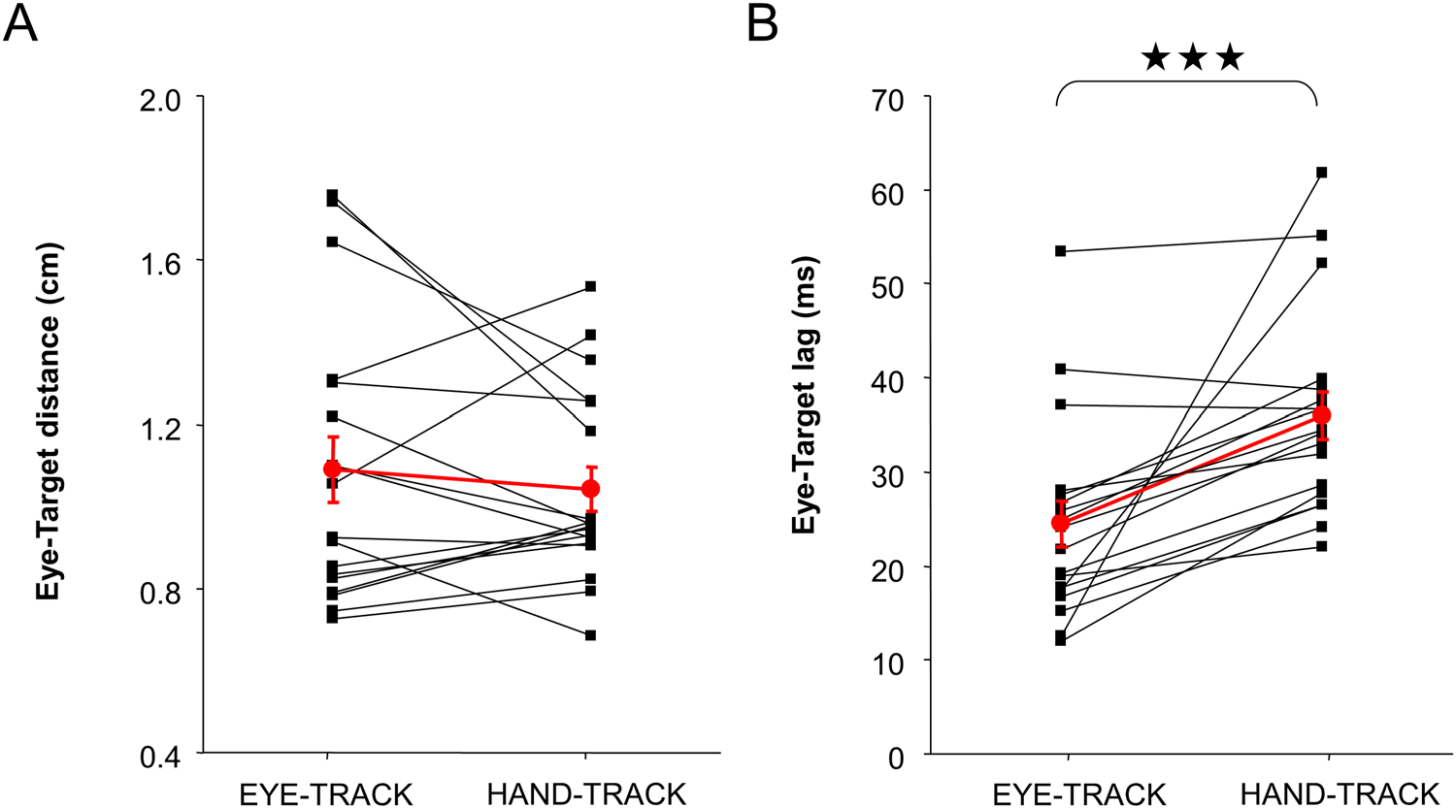
Spatial and temporal relationships between eye and target. Individual participants are represented by the black squares, and the group average is represented by the red circles. Error bars correspond to the standard error of the mean.

Overall gaze behavior in terms of eye-target distance and lag appeared rather similar in our two tasks. Regarding eye-target distance (see Fig. 5), we found no main effect of TASK (EYE-TRACK = 1.09 vs. HAND-TRACK = 1.04 cm; F(1,17) = 0.49; p > 0.55). This implies that, even though participants were not explicitly instructed to look at the target during HAND-TRACK, their gaze was as close to the target as during EYE-TRACK. The analysis of the temporal lag between the eye and target revealed a main effect of TASK (F(1,17) = 15.72; p < 0.001). However, the difference between the two tasks was rather small (EYE-TRACK = 25 vs. HAND-TRACK = 32 ms).

**Figure 6:**
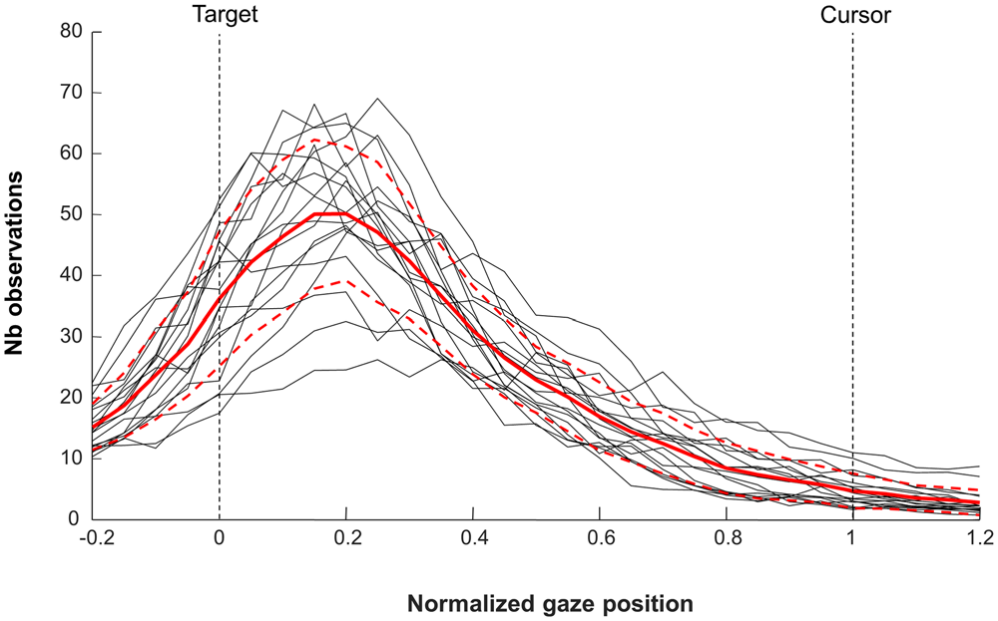
Distribution of gaze relative to the cursor and target positions during hand tracking. The mean group distribution is presented by the thick line in red (with red dotted lines indicating ±1 SD). Individual participant distributions are presented by the thin black lines.

To characterize in more detail gaze behavior during hand tracking, in Fig. 6 we present individual distributions and the mean group distribution of relative eye position with respect to the target and cursor. For all participants, distributions were unimodal with their peak much closer from the target than the cursor.

### Saccadic behavior

**Figure 7:**
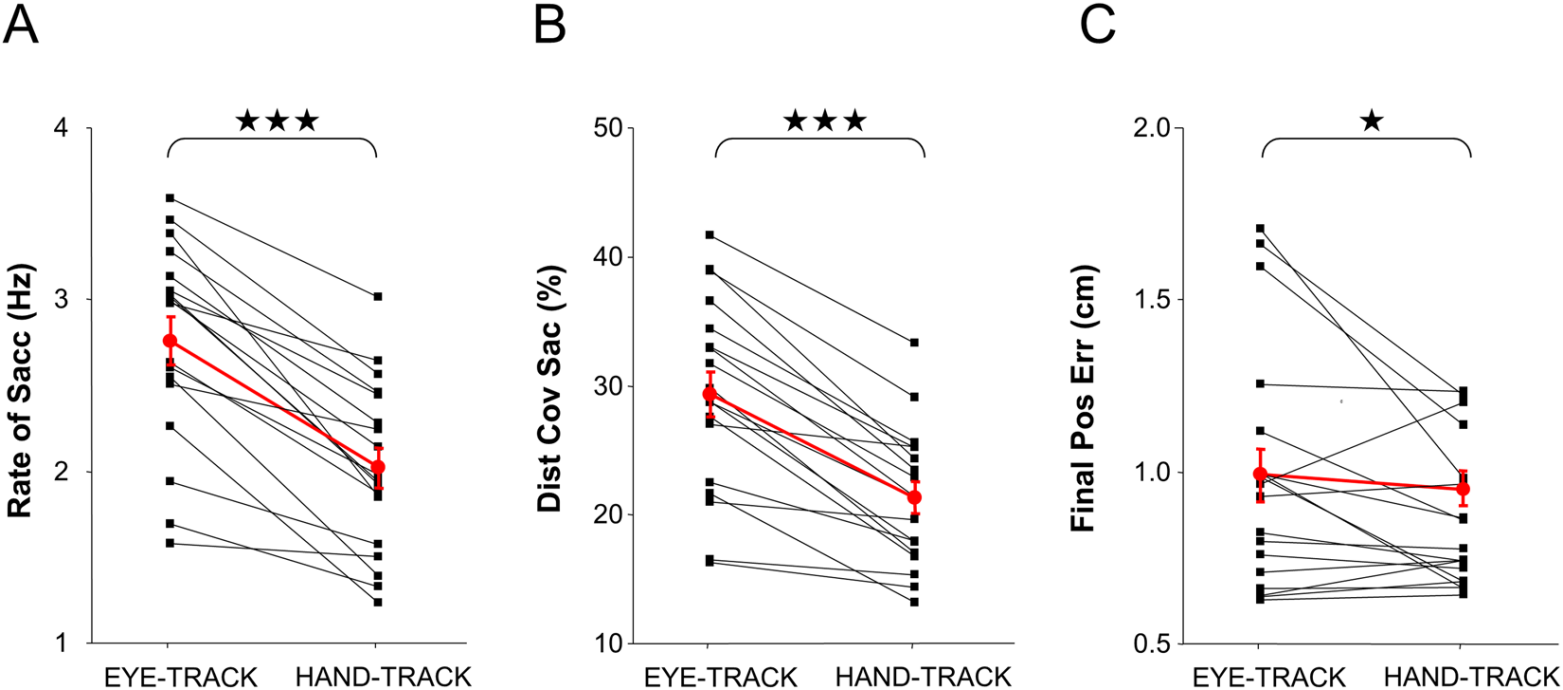
Dependent variables in relation to saccadic activity. (**A**) Rate of catch-up saccades. (**B**) Percentage of total distance covered by the eye with catch-up saccade. (**C**) Eye-target distance at the end of catch-up saccade. For all graphs, individual participants are represented by the black squares, with group average represented by the red circles. In the latter case, the error bars correspond to the standard error of the mean.

Figure 7A shows the rate of catch-up saccades in each experimental condition. Whereas participants made on average 2.76 saccades per second under EYE-TRACK, they made an average of 2.02 saccades per second under HAND-TRACK, reflecting a 27% drop (F(1,17) = 71.04; p < 0.001). However, no difference was found in terms of saccade amplitude (EYE-TRACK = 2.03 vs. HAND-TRACK = 2.00 cm; F(1,17) = 0.39; p > 0.54), or saccade duration (EYE-TRACK = 33 vs. HAND-TRACK = 32 ms; F(1,17) = 2.94; p > 0.1). In agreement with those findings we found that the contribution of catch-up saccades to total eye displacement (see Fig. 7B) was smaller during hand tracking as compared to eye tracking (HAND-TRACK = 21.3 vs. EYE-TRACK = 29.3%; F(1,17) = 63.57; p < 0.001).

Although saccades were initiated with similar positional error (EYE-TRACK = 1.57 vs. HAND-TRACK = 1.61 cm; F(1,17) = 0.42; p = 0.53), their final accuracy was greater during hand tracking (EYE-TRACK = 0.99 vs. HAND-TRACK = 0.87 cm; F(1,17) = 4.89; p < 0.05), reflecting a 13% improvement (see Fig. 7C). Overall, saccades were less frequent and slightly more accurate during hand tracking.

### Smooth pursuit behavior

**Figure 8:**
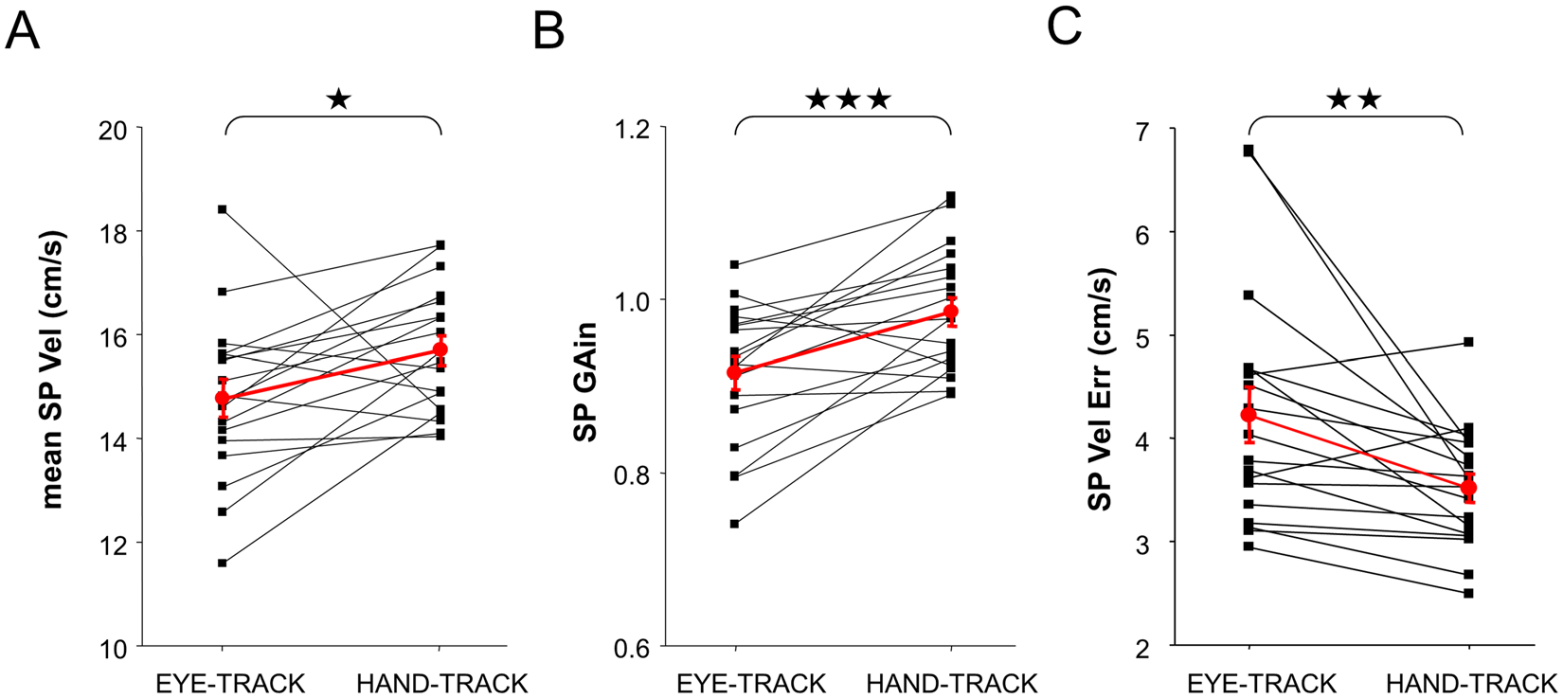
Dependent variables in relation to smooth pursuit activity. (**A**) Mean tangential velocity of smooth pursuit. (**B**) Smooth pursuit gain. (**C**) Smooth pursuit velocity error. For all graphs, individual participants are represented by the black squares, with group average represented by the red circles. In the latter case, the error bars correspond to the standard error of the mean.

Further analyses revealed that smooth pursuit (SP) was also different between eye and hand tracking (see Fig. 8). Under HAND-TRACK, in comparison to EYE-TRACK, SP velocity was 6% higher (EYE-TRACK = 14.8 vs. HAND-TRACK = 15.7 cm/s; F(1,17) = 5.52; p < 0.05), SP gain was 8% higher (EYE-TRACK = 0.92 vs. HAND-TRACK = 0.99; F(1,17) = 15.28; p = 0.001), and velocity error between eye and target decreased by 17% (EYE-TRACK = 4.23 vs. HANT-TRACK = 3.52 cm/s; F(1,17) = 9.21; p < 0.01). Overall, we conclude that SP was notably improved under hand tracking as compared to eye tracking.

### Correlation across tasks and participants

When comparing gaze behavior between the eye and hand tracking tasks, many dependent variables associated with eye movements showed a positive correlation across participants. This was the case for saccade rate (R = 0.78; p < 0.001), saccade amplitude (R = 0.70; p < 0.01), saccade duration (R = 0.55, p < 0.05), saccade accuracy (R = 0.74; p < 0.001), SP gain (R = 0.51; p < 0.05), SP velocity error (R = 0.51; p < 0.05), as well as eye-target distance (R = 0.68; p < 0.01). These correlations indicate that, in addition to an overall effect of task, variations across participants in these variables are largely independent of task. This conclusion can be appreciated from the individual participant results shown in Figs 7 and 8.

## Discussion

The main goal of this study was to test the hypothesis that gaze behavior might be driven by different strategies when tracking a moving target with the eye in comparison to the hand. We found that even though participants were not explicitly asked to track the target with the eyes during hand tracking, they kept their gaze relatively close to the target. In fact on average gaze was as close to the target as when participants were explicitly required to track the target with the eyes. However, several differences were observed in gaze behavior between the two tracking conditions. Specifically, during hand tracking, in comparison to eye tracking, catch-up saccades were less frequent and more accurate, while the gain and the accuracy of smooth pursuit were greater. In the following paragraphs we discuss these observations and their implications in more detail.

### Similarities in gaze behavior during eye and hand tracking

Our results showed that no matter whether our participants were explicitly instructed to track the target with the eyes, or with a cursor moved by the hand (in the absence of explicit requirement for gaze behavior), gaze was always directed toward the target with a similar level of accuracy. Although the average eye-target lag was slightly greater during hand tracking, no reliable difference in the average eye-target distance (about 1 cm) was observed between the tasks, suggesting similar target attractiveness for the eye in both tasks. These results correspond with earlier observations showing similar RMS error between eye and target when participants either concurrently tracked the target with eye and hand or tracked it with the eye alone^22,24^; although see^23^. To track efficiently the target with the hand, the brain needs to monitor both the position of the target and the cursor. The fact that gaze seems mostly concerned with monitoring the target suggests that the monitoring of the cursor is performed through peripheral vision and/or arm proprioception. This suggestion fits well with the idea that fixating the target improves reach accuracy because it provide retinal and extraretinal information to guide the hand^5,11,13,14^. Although such behavior has been shown for reaching to static targets and when explicitly tracking a moving target concurrently with the eye and hand, here we demonstrate it for hand tracking with free gaze (i.e., no explicit instructions about eye behavior).

### Dissimilarities in eye motion during eye and hand tracking

Despite the fact that gaze stayed similarly close to the target in both of our tasks, further analyses revealed some key differences in terms of eye motion. During hand tracking, in comparison to eye tracking, catch-up saccades were less frequent (and slightly more accurate), and the gain of smooth pursuit was higher, such that its mean velocity also increased. Overall these results support the view that gaze behavior is driven by different demands when tracking a moving target with the eye or the hand. As suggested earlier, when participants need to track a target with the hand, retinal and extraretinal information are key inputs to drive the hand. However, during saccadic eye motion, not only is retinal information unavailable due to a phenomenon known as saccadic suppression^35,36^, but gaze velocity exceeds substantially target velocity, presumably disrupting the ability to make use of extraretinal information. Thus, limiting the number of saccades, while keeping gaze reasonably close to the target and at the same speed as the target can be viewed as a strategy promoting hand tracking accuracy. This scheme resonates with the study of Mrotek and Soechting (2007) in which catch-up saccades were suppressed during a single reach to a moving target, again presumably so that retinal and extra-retinal information could be used to guide the hand during the reach^15^. However, when performing continuously hand tracking, saccades are required to keep gaze near the target. Still, by limiting the number of saccades and increasing the smooth pursuit gain (e.g., in comparison to eye tracking), the sensorimotor system can facilitate hand tracking accuracy.

It should be acknowledged that our observations regarding smooth pursuit gain and saccade frequency echo with earlier observations made when comparing eye tracking with parallel hand and eye tracking^23,24^. The fact both types of studies led to rather similar findings suggests that changes in gaze behavior seen during parallel hand and eye tracking do not arise because of dual tracking. In fact one may actually question the necessity of explicitly instructing participants to track the target with the eyes during hand tracking given that they apparently do it spontaneously. We should also note that increases in smooth pursuit gain and decreases in saccade frequency have been reported when a target is moved by the participant’s hand in comparison to when it is moved by an external agent^33,37–42^. However, in the current study the predictability of target motion cannot account for possible differences in gaze behavior, since the same externally determined target trajectories were employed in both of our tasks.

### Limitations of the study

There are several limitations in our study. First, with the KINARM setup the target was moving in depth, making adjustments in vergence mandatory for accurate eye-tracking. Because here we only measured one eye motion, we cannot state how much vergence differs across tasks. It is known that for a 3D target path, visuomotor coordination is different for the depth component (vergence) and the frontal plane^43–45^. Whether vergence is similar under our hand tracking and eye tracking tasks will need to be address by future studies. However, one study^46^ performed with a target moving only in depth indicates that, in contrast to smooth pursuit, vergence eye movements are not influenced by the presence/absence of hand movements. Second, in our task participants’ arm movements were restricted to the horizontal plane and had only two degrees of freedom (DOF), one at the shoulder and one at the elbow. In contrast, typical reaching movements recruit a larger number of DOFs^47^ and it is possible that this might influence gaze behavior. Third, our behavioural study using healthy participants cannot provide insights regarding the neural structures underlying differences in eye hand coordination between eye and hand tracking. However, structures such as the cerebellum^48^ or the parietal cortex^49^ are probable candidates that mediated the current effect since they are often evoked as crucial nodes for eye-hand coordination.

## Conclusions

Overall we conclude that tracking an externally-moved target with the eye or the hand influences significantly the saccadic and smooth pursuit system. In contrast to previous suggestions that hand motion reinforces the internal representation of target motion^23,24^, we suggest that differences in gaze behavior arises from different tasks demands. Whereas keeping gaze close to the target is important in both tasks, obtaining stable retinal and extra-retinal information is critical for guiding hand movement. More generally this interpretation fits well with the view that eye motion allows gathering relevant information for future or ongoing hand movement^1,50^.

Our results highlight the different functions that eye movements and vision play in the planning and control of limb movement, and provide an illustration of how gaze behavior emerges from the interplay between these functions and task demands. Whereas great insight into oculomotor control has been gained by studies examining eye movements in isolation^17,51–53^, examining how eye movements support the planning and control of action is important if we are to obtain a full understanding of the mechanisms underlying oculomotor control^54,55^.
